# Analysis of the Clinicopathologic Characteristics of Lung Adenocarcinoma With *CTNNB1* Mutation

**DOI:** 10.3389/fgene.2019.01367

**Published:** 2020-02-07

**Authors:** Chao Zhou, Wentao Li, Jinchen Shao, Jikai Zhao, Chang Chen

**Affiliations:** ^1^ Department of Thoracic Surgery, Shanghai Pulmonary Hospital, Tongji University School of Medicine, Shanghai, China; ^2^ Department of Thoracic Surgery, Shanghai Chest Hospital, Shanghai Jiao Tong University, Shanghai, China; ^3^ Department of Pathology, Shanghai Chest Hospital, Shanghai Jiao Tong University, Shanghai, China

**Keywords:** adenocarcinoma, lung cancer, *CTNNB1*, mutation, prognosis

## Abstract

**Introduction:**

Lung adenocarcinoma with *CTNNB1* mutation is relatively uncommon, and its clinicopathologic characteristics, disease course, and prognosis have not been well-studied.

**Methods:**

A total of 564 lung adenocarcinoma patients were enrolled in this study. The relationship between *CTTNB1* mutational status and clinicopathologic parameters, the rates of relapse-free survival (RFS) and overall survival (OS), and the mutational status of other genes commonly mutated in lung adenocarcinoma were analyzed.

**Results:**

Of 564 lung adenocarcinoma patients, 30 (5.3%) harbored *CTNNB1* mutations. Univariate analyses revealed that gender, smoking history, pleural invasion, and histological subtype were all significant predictors of RFS and OS. Pleural invasion and histological subtype remained significant predictors of RFS and OS in a multivariate analysis. There were no significant differences in RFS (*p* = 0.504) or OS (*p* = 0.054) between lung adenocarcinoma patients with *CTNNB1* mutation and those without *CTNNB1* mutation. However, patients with *CTNNB1* mutation tended to have a worse OS.

**Conclusions:**

Female patients and nonsmokers are likely to harbor *CTNNB1* mutation and primary lung adenocarcinoma with mutated *CTNNB1* has a poor prognosis.

## Introduction

Lung cancer, which has the highest incidence of all cancers and the highest rate of disease-related fatalities, is the main cause of cancer-related death worldwide (Torre et al., 2015; Gu et al., 2017a; Gu et al., 2018). Lung adenocarcinoma is the most common pathological subtype, accounting for nearly 70% of all lung tumors (Sun et al., 2010). With the introduction of low-dose computed tomography, which enables earlier detection, the incidence of lung cancer, especially early-stage lung cancer, has risen sharply in recent years (Field et al., 2012).

With the advent of genomics, molecular or genetic variants affecting disease risk can be identified (Field, 2008). Mutations in the gene encoding β-catenin (*CTNNB1*) have been detected in numerous human malignancies, including lung cancer (Woenckhaus et al., 2008), malignant mesothelioma (Shigemitsu et al., 2001), desmoid tumors (Colombo et al., 2013), colon cancer (Akyol et al., 2019), and others. Woenckhaus et al. (2008) identified a number of differentially expressed genes in smoke-exposed bronchial epithelium and nonsmall cell lung cancers (NSCLCs), they found in adenocarcinomas, the cytoplasmic expression of beta-catenin was associated with shorter survival (*p* = 0.012). Shigemitsu et al. (2001) found *CTNNB1* is infrequently mutated in lung cancer. Akyol et al. (2019) defined an immunohistochemical algorithm to dissect Wnt pathway alterations in formalin-fixed and paraffin-embedded neoplastic tissues and found all six colon adenomas of the 126 total adenomas studied for the altered/mutant β-catenin staining pattern had presumptively pathogenic point mutations or deletions in CTNNB1. The N-terminus of β-catenin, with contains conserved phosphorylated threonine/serine amino acid residues, is the most frequent location of cancer-related *CTNNB1* mutations (Dar et al., 2017). The level of free β-catenin in the cytoplasmic pool is regulated by ubiquitination and proteasomal degradation (Akyol et al., 2019). β-catenin is a member of the Wnt signaling cascade and is associated with cadherin-mediated cell–cell adhesion systems (Woenckhaus et al., 2008). In lung tumors, the immunohistologic loss of β-catenin membrane staining along with a corresponding increase cytoplasmic or nuclear staining has been reported (Nozawa et al., 2006).

Although *CTNNB1* mutation occurs in many tumors types, it has not been well-studied in the context of lung adenocarcinoma, and the clinicopathologic characteristics and prognosis of lung adenocarcinoma with mutated *CTNNB1* has not been described. Therefore, we compared the clinicopathologic characteristics of 30 lung adenocarcinomas with *CTNNB1* mutations with those of 534 lung adenocarcinomas with wild-type *CTNNB1*.

## Materials and Methods

From July 2008 to April 2013, resected primary lung adenocarcinomas were collected at the Department of Thoracic Surgery of Shanghai Chest Hospital, Shanghai Jiaotong University. To confirm the diagnosis of primary lung cancer, all the patients received thorough preoperative testing at our hospital, including physical exams, serological tests, pulmonary function tests, chest/brain computed tomography (CT), technetium bone scanning, and abdominal ultrasound. Biopsies were done by bronchoscopy or endobronchial ultrasound-guided transbronchial needle aspiration, and in some cases, positron emission tomography CT was used to exclude mediastinal lymph node metastases (Gu et al., 2017b). The lung adenocarcinoma subtype was determined by light microscopy intraoperatively, using frozen sections, and confirmed postoperatively, using paraffin-embedded sections. All surgical samples had at least 5% tumor content. Each case was reviewed by at least two junior pathologists and a senior pathologist to confirm the histologic subtype of resected lung neoplasms. The combination of routine preoperative examination and intra-/postoperative pathological diagnosis is recommended to make an exact lung cancer diagnosis.

In total, 601 patients with primary lung adenocarcinoma were identified. Of these, 17 and 20 patients were excluded because they received neoadjuvant chemotherapy or were lost to follow-up, respectively. The remaining 564 patients were enrolled in this study.

Informed consent was given by all patients or their legal representatives. The study was initiated after obtaining Institutional Review Board approval. The medical records for all patients were reviewed to collect corresponding clinicopathologic data, including sex, age, smoking status, pathologic tumor, node, and metastasis (TNM) stage [according to the staging system of the 7th edition of the American Joint Committee on Cancer (Edge et al., 2010)], thyroid transcription factor-1 status, and treatment information. Data on disease recurrence and survival were obtained from follow-up clinic visits or by telephone.

### Bioinformatics Analysis

Data of The Cancer Genome Atlas (TCGA) were analyzed by Gene Expression Profiling interactive Analysis (http://gepia.cancer-pku.cn/) and Kaplan–Meier Plotter (http://kmplot.com/analysis/index.php?p=service&cancer=lung). Gene *CTNNB1* were further analyzed by Gene Expression Profiling interactive Analysis and the survival curves were draw and compared by Kaplan–Meier Plotter.

### Mutational Analysis

The mutational status of *EGFR*, *KRAS*, and *CTNNB1* was determined by targeted sequencing and verified by DNA sequencing analysis. Relevant primers were designed to amplify all known *ALK* fusion variants by quantitative real-time reverse transcriptase PCR of cDNA. *ALK* fluorescent *in situ* hybridization was used to confirm the presence of *ALK* gene fusions (Wang et al., 2012).

### Statistical Analysis

Clinicopathologic data was analyzed using the SPSS 22.0 software package (SPSS Inc, Chicago, IL). Relapse-free survival (RFS) and overall survival (OS) were estimated by the Kaplan–Meier method, and differences were compared by log-rank testing using Prism 6 (GraphPad Software, La Jolla, CA). A *p* value of <0.05 was considered statistically significant.

## Results

### Mutational Status of Lung Adenocarcinomas

**Figure 1:**
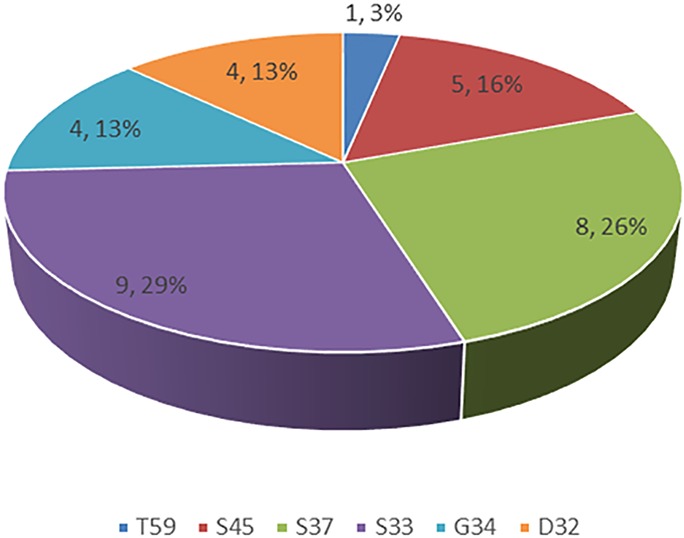
Spectrum of CTNNB1 mutations in lung adenocarcinoma.

Of the 564 lung adenocarcinoma patients examined, 30 (5.3%) harbored *CTNNB1* mutations (**Table 1**). The distributions of specific mutation types are shown in **Figure 1**.

**Table 1:** Characteristics of lung adenocarcinoma with CTNNB1 mutation.

Cases	Gender	Age	Smoking	Subtype	Tumor size (cm)	Stage	CTNNB1 mutation	RFS (months)	OS (months)
1	F	57	Never smoker	A + P	3	2a	S45F	35.4	46.8
2	F	52	Never smoker	S + P	3	3a	S45F	6.3	26.1
3	F	60	Never smoker	A + P	4.1	3a	D32Y	46.5	82+
4	F	59	Never smoker	P	2.8	3a	D32Y	3.6	22.3
5	F	44	Never smoker	S + A	3	3a	D32Y	22	56+
6	F	49	Never smoker	P + S + L	8.4	3a	S33C	3.2	16.8
7	M	59	Never smoker	L + A	1.9	1a	S37A	25.4	47+
8	M	65	Smoker	P	4.6	3a	S33C	25	68+
9	M	62	Smoker	P	2.4	1a	S37F	45+	45+
10	F	55	Never smoker	A + P	5	1b	S45P	12	43+
11	M	59	Smoker	IMA	5	3a	G34V	2.4	19
12	F	75	Never smoker	P + M	2.1	1a	S33Y	45+	45+
13	F	60	Never smoker	A + P	1.6	1b	S33C	63+	63+
14	F	74	Never smoker	P + M	4.3	1b	S37C	63+	63+
15	M	67	Smoker	A + P + M	2.1	2b	S37C	3.2	44
16	F	69	Never smoker	A + P + M	2.9	3a	S37F	16.8	29
17	F	70	Never smoker	A + M	1.7	1a	D32H	56+	56+
18	F	62	Never smoker	S + P	2.1	1b	S33F	6.4	10
19	F	55	Never smoker	A + L	2.8	3a	S37F	58+	58+
20	M	41	Never smoker	A + P	2.6	1a	S33C	62+	62+
21	F	59	Never smoker	P + A	4.5	1b	S37F	60+	60+
22	F	68	Never smoker	P + A + M	4.3	1b	G34R	4.8	13.8
23	F	72	Never smoker	S + P	2.1	1a	S45F	54+	54+
24	F	68	Never smoker	A + P	2.9	1b	S33C	19	29.4
25	F	59	Never smoker	A + P + M	2.4	1a	S33C	16	34.2
26	M	46	Never smoker	A + P	2.6	1a	G34R	15	35
27	F	70	Never smoker	P + A	2.1	1b	S37C	19.6	35.3
28	M	74	Never smoker	M	5.6	2a	S45P	56+	56+
29	F	60	Never smoker	A + S	3.8	1b	S33C	48+	48+
30	M	61	Smoker	P + M	4.6	1b	G34V	61+	61+

### Relationship Between Clinicopathologic Factors and *CNTTB1* Mutational Status

Of the 30 patients with *CTNNB1* mutations, there were 21 (70%) female patients and 9 (30%) male patients, ranging in age from 22 to 81 years (median, 59 years). Histologically, 263 of the tumors were acinar-predominant (47%), 115 were micropapillary-predominant (20%), 94 were papillary-predominant (17%), 49 were lepidic-predominant (9%), 32 were mucinous adenocarcinoma-predominant (5%), and 11 were solid-predominant (2%). Most of the patients had early-stage lung cancer in both the *CNTTB1* mutation group (stage I: 18/30, 60%) and the *CNTTB1* wild-type group (stage I: 241/534, 45%). Age (*p* = 0.851), tumor size (*p* = 0.256), lymph node status (*p* = 0.184), pathologic stage (*p* = 0.322), and the presence of pleural invasion (*p* = 0.459) were similar between lung adenocarcinomas with *CTNNB1* mutation and lung adenocarcinomas without *CTNNB1* mutation, but the former group tended to have more female patients (*p* < 0.001) and more smokers (*p* = 0.019) (**Table 2**).

**Table 2:** Features of patients with lung adenocarcinoma harboring *CTNNB1* mutations.

	CTNNB1 mutation		CTNNB1 wild type		
	No.	Percent	No.	Percent	*p* value
**Total**	30	5.3%	534	94.7%	
**Sex**					
Male	9	30%	259	48.5%	
Female	21	70%	275	51.5%	<0.001
**Age**					
≥60 years	14	47%	234	44%	
<60 years	16	53%	300	56%	0.851
**Smoking status**					
Smoker	5	17%	332	62%	
Never-smoker	25	83%	202	38%	0.019
**Tumor size**					
≤3c m	21	70%	314	59%	
>3 cm	9	30%	220	41%	0.256
**Lymph Node status**			
N0	21	70%	300	56%	
N1/2	9	30%	234	44%	0.184
**Pathologic stage**					
I	18	60%	241	45%	
II	3	10%	81	15%	
III	9	30%	187	35%	
IV	0	/	25	5%	0.322
**Pleural invasion**					
0	15	50%	299	56%	
1/2	15	50%	235	44%	0.459
**Pathological subtype**					
Lepidic	1	3%	48	8%	
Acinar	13	43%	250	47%	
Papillary	10	33%	84	16%	
Micropapillary	4	13%	111	21%	
Solid	1	3%	10	2%	
IMA	1	3%	31	6%	0.168
**TTF1**					
Positive	16	53%	339	63%	
Negative	14	47%	195	37%	0.331
**EGFR**					
Present	21	70%	314	59%	
Absent	9	30%	220	41%	0.256
**KRAS**					
Present	1	3.3%	56	10.5%	0.347
Absent	29	96.7%	478	89.5%	
**ALK**					
Present	2	7%	28	5%	
Absent	28	93%	506	95%	0.669

### Relationship Between *CNTTB1* Mutational Status and Survival

Univariate analysis revealed that gender, smoking history, pleural invasion, and histological subtype were all significant predictors of RFS and OS (**Table 3**). Pleural invasion and histological subtype were still significant predictors of RFS and OS in a multivariate analysis (**Table 4**).

**Table 3:** Independent predictors of overall survival.

Univariate analysis	HR	95% CI	*p* value
Gender, male vs. female	1.706	1.194–2.438	0.003
Age	0.988	0.971–1,006	0.18
Smoke, never vs. ever	1.464	1.025–2.09	0.036
Pleural invasion, yes vs.no	0.671	0.4–0.814	0.002
Subtypes			
Lepidic	0.041	0.004–0.479	0.011
Acinar	0.801	0.561–1.142	0.801
Papillary	0.927	0.574–1.497	0.757
Micropapillary	0.438	0.061–3.134	0.411
Solid	2.918	2.021–4.213	0.0001
Invasive mucinous	0.726	0.267–1.97	0.529
EGFR mutation, no vs. yes	0.746	0.523–1.065	0.106
ALK, negative vs. positive	1.411	0.689–2.89	0.347
CTNNB1 mutation, yes vs.no	1.746	0.982–3.103	0.058
Multivariate analysis	HR	95% CI	*p* value
Gender, male vs. female	1.995	1.183–3.367	0.01
Age	0.991	0.974–1.009	0.341
Smoke, never vs. ever	0.769	0.449–1.318	0.339
Pleural invasion, yes vs.no	0.8	0.668–0.957	0.015
Subtypes			
Lepidic	0.001	/	0.949
Acinar	1.321	0.456–3.826	0.608
Papillary	1.344	0.431–4.188	0.611
Micropapillary	0.641	0.067–6.13	0.7
Solid	3.247	1.117–9.439	0.031
Invasive mucinous	/	/	/
EGFR mutation, no vs. yes	1.14	0.745–1.744	0.547
ALK, negative vs. positive	1.494	0.665–3.358	0.331
CTNNB1 mutation, yes vs.no	1.784	0.981–3.247	0.058

**Table 4:** Independent predictors of relapse-free survival.

Univariate analysis	HR	95% CI	*p* value
Gender, male vs. female	1.706	1.194–2.438	0.003
Age	0.988	0.971–1,006	0.18
Smoke, never vs. ever	1.464	1.025–2.09	0.036
Pleural invasion, yes vs.no	0.671	0.4–0.814	0.002
Subtypes			
Lepidic	0.041	0.004–0.479	0.011
Acinar	0.801	0.561–1.142	0.801
Papillary	0.927	0.574–1.497	0.757
Micropapillary	0.438	0.061–3.134	0.411
Solid	2.918	2.021–4.213	0.0001
Invasive mucinous	0.726	0.267–1.97	0.529
EGFR mutation, no vs. yes	0.746	0.523–1.065	0.106
ALK, negative vs. positive	1.411	0.689–2.89	0.347
CTNNB1 mutation, yes vs.no	1.746	0.982–3.103	0.058
Multivariate analysis	HR	95% CI	*p* value
Gender, male vs. female	1.127	0.76–1.673	0.552
Age	0.995	0.988–1.007	0.435
Smoke, never vs. ever	1.435	0.957–2.15	0.081
Pleural invasion, yes vs.no	0.78	0.692–0.88	< 0.001
Subtypes			
Lepidic	0.345	0.145–0.822	0.016
Acinar	0.997	0.54–1.839	0.992
Papillary	0.967	0.497–1.881	0.92
Micropapillary	1.45	0.516–4.077	0.481
Solid	1.731	0.929–3.224	0.084
Invasive mucinous	/	/	/
EGFR mutation, no vs. yes	1.19	0.889–1.592	0.243
ALK, negative vs. positive	1.159	0.641–2.095	0.626
CTNNB1 mutation, yes vs.no	1.206	0.737–1.974	0.457

**Figure 2:**
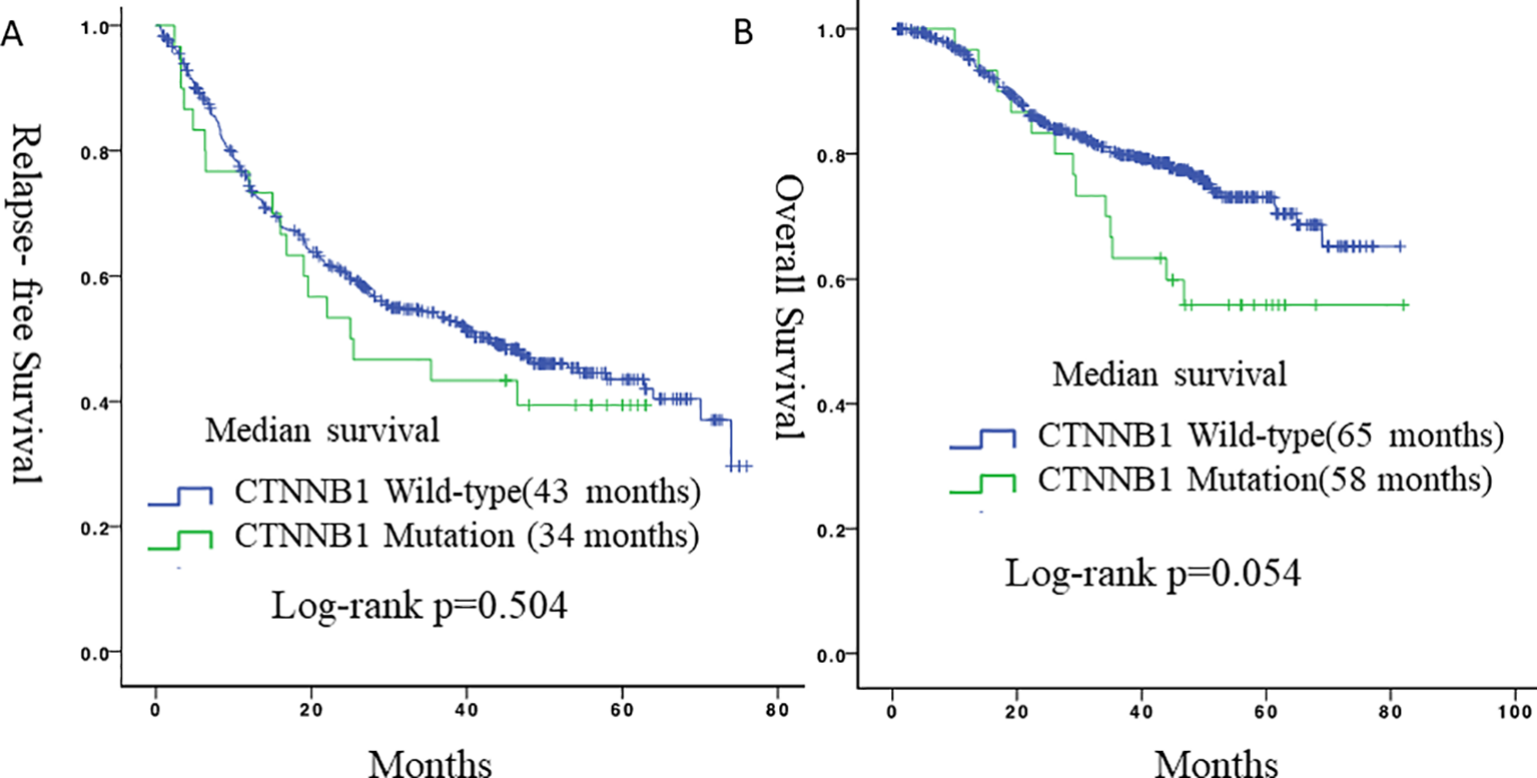
Survival curves for relapse-free survival and overall survival according to CTNNB1 status. **(A)** Relapse-free survival between the two groups. **(B)** Overall survival between the two groups.

During follow-up, 19 (63.3%) patients with lung adenocarcinomas with mutated *CTNNB1* and 259 (48.5%) patients with lung adenocarcinomas with wild-type *CTNNB1* experienced a relapse, and 10 (33.3%) and 111 (20.8%) patients died, respectively. There were no statistically significant differences in RFS (*p* = 0.504) or OS (*p* = 0.054) between patients with *CTNNB1* mutation and patients without *CTNNB1* mutation (**Figure 2**). However, patients with *CTNNB1* mutation tended to have a worse OS.

As for lung adenocarcinomas from TCGA, there was no significant differences in the distributions of CTNNB1 mRNA expression among different lung adenocarcinoma stages (**Supplementary Figure 1**). Besides, between lung adenocarcinoma patients with and without CTNNB1 mutation, there was no significant differences in RFS (*p* = 0.49), while significant differences were found in OS (*p* = 8.9e−05). (**Supplementary Figures 2** and **3**).

## Discussion

Lung cancer remains the leading cause of cancer-related death worldwide (Gu et al., 2017). Low-dose computed tomography screening reduces the mortality of lung cancer by as much as 20% in high-risk patients (National Lung Screening Trial Research Team, 2011). Early detection and diagnosis increases the number of patients who are newly diagnosed with lung cancer while it is still early-stage, improving the prognosis of lung cancer patients as a whole. For individuals at a high risk of developing lung cancer, periodic screening could have a survival benefit. Recently, some lung cancer risk prediction models have been constructed to make lung cancer screening more efficient (Spitz et al., 2007; Raji et al., 2012). With the development of gene mutation testing, targeted therapy has changed the treatment strategy for lung cancer. In this study, we describe the clinicopathological characteristics of lung adenocarcinoma with *CTNNB1* mutation.

β-catenin is important for the establishment and maintenance of the epithelial layer and is a key downstream component of the canonical Wnt signaling pathway. The WNT/β-catenin pathway is involved in cancer and pluripotent stem cell signaling, which may suggest the mechanism underlying cancer stem cells. In this study, out of 564 patients, 30 (5.3%) patients with *CTNNB1* mutations were identified. Kase et al. (2000) conducted an immunohistochemical analysis of 331 lung cancer specimens and reported that β-catenin expression was reduced in 122 (37%) of the samples, which was associated with significantly worse patient survival. Similarly, Woenckhaus et al. (2008) reported that reduced membrane staining of β-catenin and its abnormal accumulation in the cytoplasm and/or nuclei of lung adenocarcinoma cells was associated with shorter survival (*p* = 0.012). Another study also suggested that reduced β-catenin expression in surgically resected non-small cell lung cancer specimens was associated with lymph node metastasis and a poor prognosis (Retera et al., 1998). These studies suggest that decreased expression of β-catenin is associated with an unfavorable prognosis in lung cancer.

In our study, during follow-up, 19 patients (63.3%) with lung adenocarcinomas with *CTNNB1* mutations and 259 patients (48.5%) with lung adenocarcinomas with wild-type *CTNNB1* relapsed, and 10 (33.3%) and 111 (20.8%) patients died, respectively. Patients with *CTNNB1* mutations therefore tended to have a worse prognosis, although this did not reach statistical significance. When compare with data from TCGA, patients with CTNNB1 mutation in TCGA also had worse OS. Our findings therefore correspond well to the results of previous studies and common directory (Sunaga et al., 2001).

In Cox proportional hazards models, univariate analyses revealed that gender, smoking history, the presence of pleural invasion, and histological subtype were all significant predictors of RFS and OS. Pleural invasion and histological subtype remained significant predictors of RFS and OS in a multivariate analysis. With respect to histological subtype, adenocarcinoma patients with micropapillary or solid subtypes, which are defined as high-risk subtypes in the 2011 classification proposed by the International Association for the Study of Lung Cancer/American Thoracic Society/European Respiratory Society (Travis et al., 2011), had significantly worse prognosis. As for pleural invasion, pleural invasion, as well as visceral invasion, is considered an aggressive and invasive factor in NSCLC and has been included in the TNM staging system as a factor that should upstage the T factor (Rami-Porta et al., 2007; Travis et al., 2008; Butnor and Travis, 2012). Shimizu et al. (2005) demonstrated that velopharyngeal insufficiency (VPI) is a significant and independent predictor of a poor prognosis regardless of tumor size or N status, and as a result, VPI is a good indicator of the degree of invasion and aggressiveness of NSCLC. As more early-staged lung neoplasms are detected, whether VPI has impact on survival of patients with early-staged lung cancer is unknown. Therefore, Jiang et al. (2015) published a meta-analysis and found VPI together with tumor size has a synergistic effect on survival in patients with N0 disease. Patients with stage IB NSCLC and larger tumor size with VPI might be considered for adjuvant chemotherapy after surgical resection and need careful preoperative evaluation and postoperative follow-up.

There are several limitations to this study. First, the sample size was relatively small. Contributing to the small sample size, there were several patients with *CTNNB1* gene mutations who could not be included in the data analysis because of incomplete clinicopathological records. Finally, the patients’ outcomes could have been influenced by the use of different treatment strategies, which may confound the survival analysis.

In summary, our results suggest that female patients and nonsmokers are likely to harbor *CTNNB1* mutation and primary lung adenocarcinoma with mutated *CTNNB1* has a poor prognosis. Further research is needed to verify our results. However, these data suggest that β-catenin could be a potential therapeutic target for advanced-stage lung cancer.

## Data Availability Statement

The raw data supporting the conclusions of this article will be made available by the authors, without undue reservation, to any qualified researcher.

## Ethics Statement

The studies involving human participants were reviewed and approved by Ethics committee of Shanghai chest hospital. The patients/participants provided their written informed consent to participate in this study.

## Author Contributions

Conception and design: CZ and CC. Development of methodology: CZ, WL, and CC. Acquisition of data (provided surgical samples, gene detection, pathological diagnosis, acquired and managed patients, provided facilities, etc.): CZ, WL, JS, JZ, and CC. Analysis and interpretation of data: CZ. Writing, review, and/or revision of the manuscript: CZ and CC.

## Funding

This work was supported by Shanghai Pulmonary Hospital Innovation Team Program (fkcx1906).

## Conflict of Interest

The authors declare that the research was conducted in the absence of any commercial or financial relationships that could be construed as a potential conflict of interest.
