# Supplementary figures and images for: Analysis of the Clinicopathologic Characteristics of Lung Adenocarcinoma With CTNNB1 Mutation
Source: Front Genet. 2020 Feb 7;10:1367. doi: 10.3389/fgene.2019.01367 (PMC7026668; doi:10.3389/fgene.2019.01367)

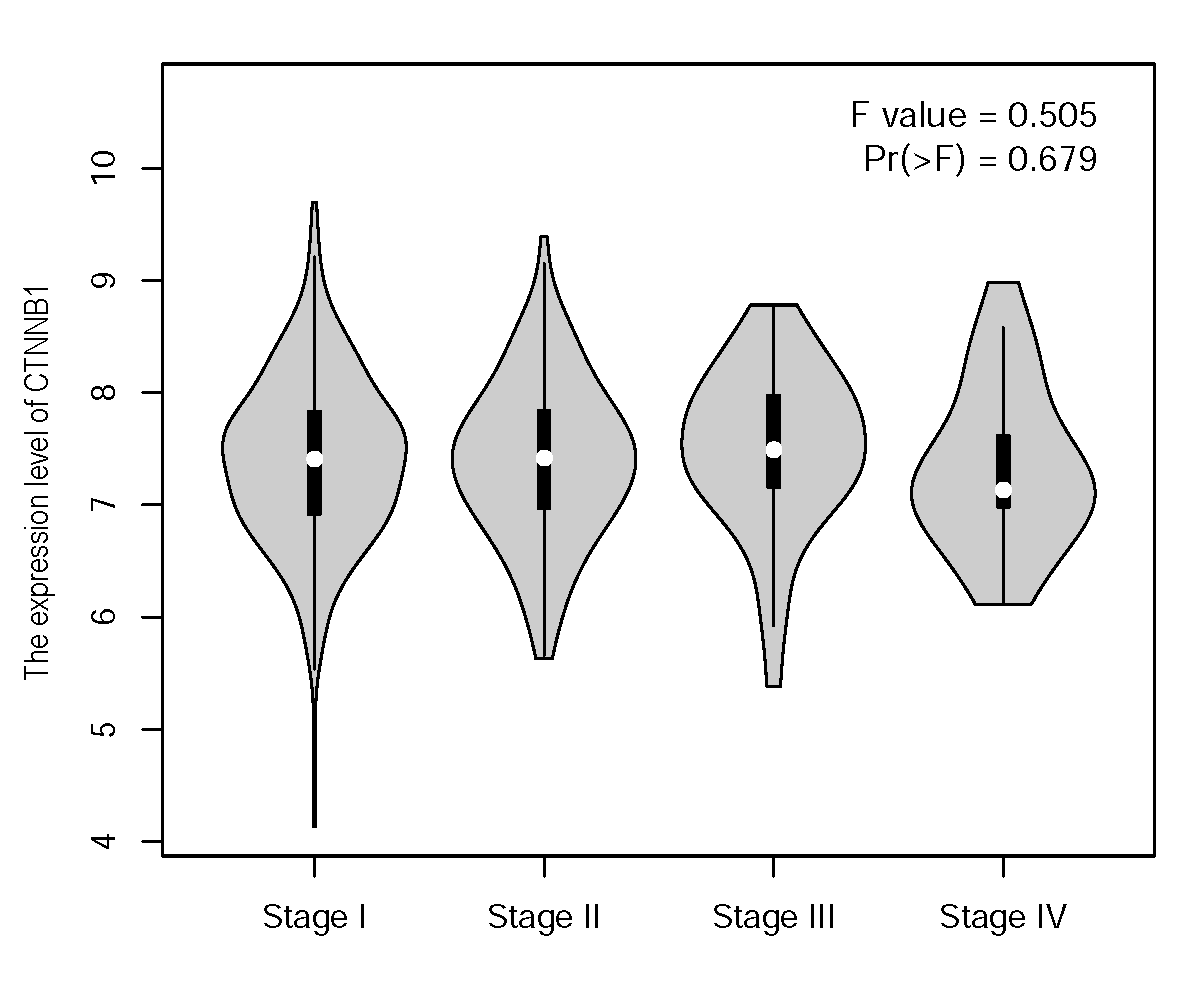

Supplement: Supplementary file 1 [file Image_1.tiff]

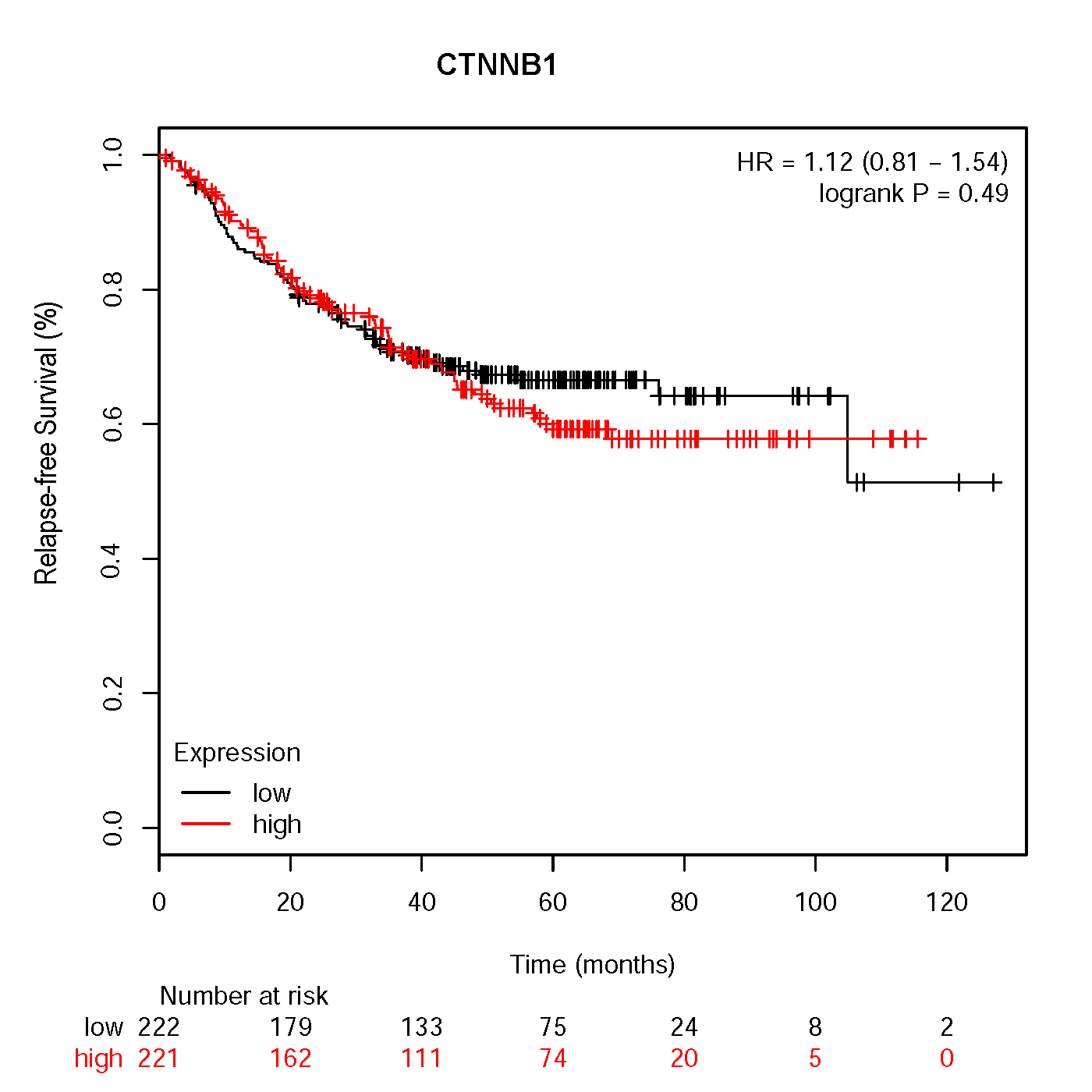

Supplement: Supplementary file 2 [file Image_2.tif]

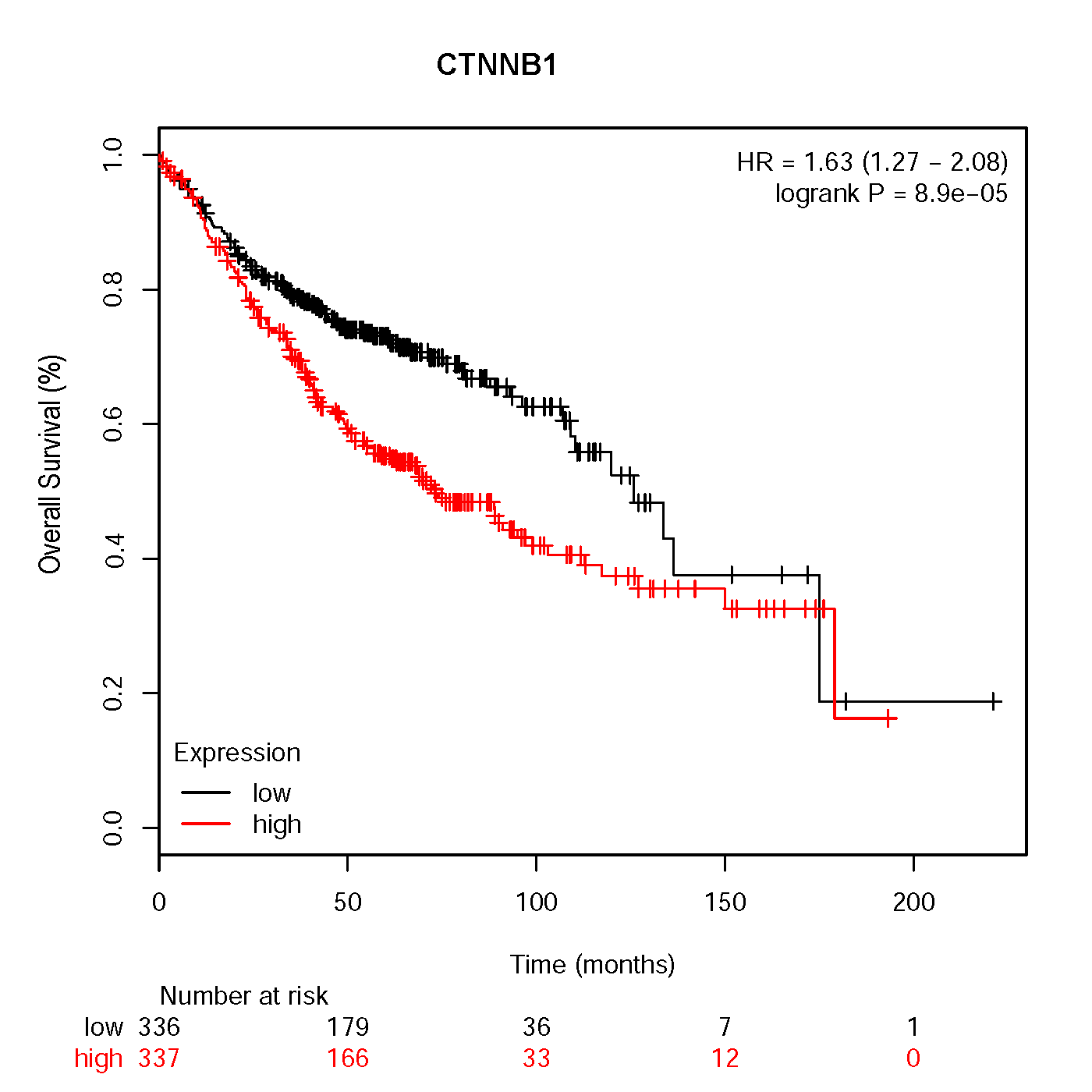

Supplement: Supplementary file 3 [file Image_3.tif]
